# Beyond Ejection Fraction: RV Function and Diastolic Markers as Predictors of Kidney Disease

**DOI:** 10.1111/echo.70378

**Published:** 2025-12-28

**Authors:** Elettra Pomiato, Biagio Castaldi, Giovanni Di Salvo

**Affiliations:** ^1^ Department of Women's and Children's Health University of Padua Padua Italy

**Keywords:** cardiorenal syndrome, chronic kidney disease (CKD), echocardiography, heart failure with reduced ejection fraction (HFrEF), right ventricular dysfunction

1

Heart failure (HF) is one of the leading health‐care problems, and despite progress in medical management, devices, and advanced therapies, the prognosis of affected patients remains poor. Among patients with HFrEF, chronic kidney disease (CKD) represents one of the most common comorbidities and, when present, confers the highest population‐attributable risk for all‐cause mortality and heart failure hospitalization among all comorbid conditions.

The recent article by Al‐Rubai et al. elegantly discusses echocardiographic predictors of kidney disease in a cohort of adult patients with HFrEF. Although retrospective, this study involved a large cohort of patients (>1100) with roughly 10 years of follow‐up. Unsurprisingly, the cumulative prevalence of CKD in this study was as high as 25%, along with a high frequency of diabetes mellitus (DM) and hypertension [1].

HF, DM, and CKD are common and interlinked conditions, yet the cross‐talk between CKD and HF remains one of the most clinically challenging intersections in modern medicine. The term cardiorenal syndrome and its classification describe this bidirectional interplay, in which dysfunction of one organ adversely affects the other through hemodynamic, neurohormonal, and metabolic mechanisms. Importantly, cardiac and renal diseases have many pathophysiological pathways in common, including inflammatory and direct cellular immune‐mediated mechanisms, neurohormonal responses, metabolic and nutritional deviations, altered hemodynamic and acid–base or fluid status, and impaired erythropoiesis [2].

According to the authors’ evaluation, left ventricular ejection fraction (LVEF), tricuspid annular plane systolic excursion (TAPSE), and E/e′ ratio were independent predictors of incident CKD in multivariate regression analysis. LVEF, as a prognostic marker in HF, has been extensively evaluated over the past decades, and its role as a risk factor is well established, although this becomes less obvious in patients with more severe ventricular dysfunction, and its correlation with functional capacity or quality of life is debated [3]. Preoperative LVEF is also an independent risk factor for acute kidney injury (AKI) after noncardiac surgery [4].

From a pathophysiological perspective, LVEF has traditionally been identified as the initial driver of renal dysfunction, with low cardiac output being the major determinant of renal hypoperfusion, namely pre‐renal CKD, while the right ventricle was considered related only to the lungs. In contrast, emerging evidence increasingly indicates that right ventricular (RV) dysfunction and congestion may play a far more important role in the development of CKD in patients with HFrEF. RV failure results in elevated right atrial pressure and a subsequent increase in renal venous pressure. Elevated venous pressure reduces the renal perfusion gradient, impairs renal microcirculation, and increases interstitial edema within the noncompliant renal capsule, resulting in congestive nephropathy and ultimately leading to reduced glomerular filtration independent of cardiac output [5].

Although it is now recognized that RV dysfunction can contribute to renal impairment, the challenge lies in identifying reliable markers of RV dysfunction. The authors identified TAPSE as an independent predictor of CKD. TAPSE is an easily obtainable and reproducible echocardiographic parameter, and in a previous study, TAPSE ≤14 mm was associated with eGFR <60 mL/min/1.73 m^2^ (OR [95% CI] = 2.51 [1.44–4.39], *p* < 0.0001), as well as an independent risk factor for all‐cause mortality in a cohort of patients with HFrEF [6]. However, the role of TAPSE remains debated, as other studies extensively investigating RV function by echocardiography suggested RV myocardial work index or RV ventricular–arterial coupling as better predictors of RV dysfunction and related hospitalizations [7, 8].

The E/e′ ratio was the third identified independent predictor of CKD in the study. LV diastolic dysfunction is characterized by impaired LV filling and represents a strong predictor of cardiovascular events and HF. The major determinant of LV diastolic dysfunction is hypertension, which was highly prevalent in the cohort [1], while additional factors include age, ethnicity, dietary sodium intake, obesity, diabetes mellitus, and CKD, highlighting once again the complex interplay among various risk factors in promoting HFrEF [9]. Diastolic dysfunction is a well‐recognized risk factor for CKD, and in another recent study, a septal E/e′ ratio of ≥12 predicted renal events [10].

Importantly, the presence of CKD is critical in determining guideline‐directed medical therapy for HFrEF and is the most common reason for failure to uptitrate therapy, placing patients with both CKD and HFrEF at risk of worse prognosis and inadequate medical treatment [11]. Among novel therapies in patients with HFrEF, the only two with strong evidence for reducing cardiovascular death and hospitalization in patients with CKD Stage ≥3B are vericiguat and SGLT2 inhibitors.

Vericiguat is a soluble guanylyl cyclase stimulator that, in the VICTORIA study, significantly decreased cardiovascular death or hospitalization for heart failure in patients with HFrEF who remained symptomatic despite optimal medical therapy. Thanks to its pharmacodynamic properties, vericiguat potentiates vasodilation, lowers platelet aggregation, and provides protection against endothelial dysfunction, inflammation, oxidative stress, and ultimately myocardial fibrosis. Clinically, vericiguat acts on both the left and the right heart, promoting LV remodeling and LVEF as well as improving RV ventricular–arterial coupling [7, 12]. Based on its pharmacodynamics, it could be hypothesized that it would improve or maintain GFR in patients with HFrEF; yet, in the VICTORIA study, there was a decrease in eGFR compared with placebo, though this was not statistically significant after 48 weeks of treatment. Notably, because patients with CKD up to Stage 4 were included in the trial, vericiguat can be administered in these patients, and no increase in adverse events was observed compared to placebo [13].

SGLT2 inhibitors are a relatively new class of medications for HFrEF that, among other effects, decrease glucose reabsorption in the renal tubules, leading to increased glucose and sodium excretion. They have been demonstrated to be safe and effective, improving clinical outcomes in three major randomized trials involving patients with HFrEF. SGLT2 inhibitors lower the risk of composite renal outcomes in patients regardless of whether they had CKD at baseline. In DAPA‐HF, treatment with SGLT2 inhibitors reduced serious renal events compared with placebo, and serious adverse events were less common with dapagliflozin than with placebo in patients with CKD [14]. Similar results were observed for empagliflozin in EMPEROR‐HF, which also included patients with Stage 4 CKD [15].

In conclusion, CKD is prevalent among patients with HFrEF, and although the presence of CKD is a recognized risk factor for mortality and morbidity, the complex interplay between cardiac and renal dysfunction—also influenced by Type 2 diabetes and hypertension—remains incompletely understood despite extensive investigation. Medical treatment in these patients remains suboptimal, although SGLT2 inhibitors and vericiguat have been shown to be safe even in patients with Stage 4 CKD.

## Funding

The authors have nothing to report.
